# Exploration of Hypolipidemic Effects of Sterols from *Pleurotus tuber-regium*(Fr.) Sing Sclerotium

**DOI:** 10.3390/foods14142498

**Published:** 2025-07-16

**Authors:** Chao Wang, Yuan Liu, Yuting Duan, Haiping Lin

**Affiliations:** 1 College of Food and Health, Zhejiang A&F University, Hangzhou 311300, China; liuyuan3825@stu.zafu.edu.cn (Y.L.); 19857044021@163.com (Y.D.); 2 State Key Laboratory of Subtropical Silviculture, Zhejiang A&F University, Hangzhou 311300, China

**Keywords:** *Pleurotus tuber-regium*(Fr.) sing sclerotium, sterol, extraction technology, hyperlipidemia

## Abstract

The extraction technology of sterol was confirmed by ethanol reflux and saponification in this study. The orthogonal test was employed to assess the impact of extraction time, solid–liquid ratio, ethanol concentration and extraction temperature on the yield of sterol extraction. Hyperlipidemia model mice were established by feeding a high-fat and -sugar diet, and different doses of sterol extracts were given to the mice by gavages. The optimal extraction conditions were identified as an extraction time of 80 min, a solid–liquid ratio of 1:10, an ethanol concentration of 95%, and an extraction temperature of 90 °C, resulting in a sterol concentration of 1.16 mg/g. Compared with the high-fat model group, the high-dose group significantly reduced body weight by 17.2%, liver weight by 30.9%, and serum low density lipoprotein cholesterol by 20.0% (*p* < 0.05), while serum total cholesterol (5.59 ± 0.48 vs. 5.68 ± 0.64 mmol/L) and high-density lipoprotein cholesterol (0.98 ± 0.05 vs. 0.93 ± 0.03 mmol/L) showed no significant changes compared to the model group.

## 1. Introduction

Hyperlipidemia has become a serious disease affecting human health in modern society [1,2,3]. Hyperlipidemia means that the lipid content in the blood exceeds the normal range. Blood lipids refer to lipid substances contained in plasma, including triglyceride (TG), total cholesterol (TC), monoglyceride, diglyceride, phospholipids, cholesteryls and non-esterified fatty acid. Among them, the contents of serum triglyceride (TG), total cholesterol (TC), low-density lipoprotein cholesterol (LDL-C) and high-density lipoprotein cholesterol (HDL-C) are closely related to human health and are important indicators for measuring blood lipid levels in the human body. Heightened concentrations of TG, TC, and LDL-C, coupled with diminished levels of HDL-C, are predisposing factors that can expedite vascular adhesion and constriction of blood vessels. Consequently, this process can give rise to atherosclerosis, coronary artery disease, hypertension, cerebral infarction, and an array of associated pathologies, posing grave risks to human wellbeing. Moreover, the age of onset is showing a trend of younger ages. Therefore, the treatment of hyperlipidemia has attracted great attention from the global medical community.

Lipid metabolism is a complex process in the human body, including the synthesis, storage, and breakdown of TG, fatty acids, and cholesterol. This process is regulated and influenced by multiple pathways of genetics, hormones, and various enzymes [4]. TG is a physiologically normal metabolic substance in the cardiovascular system, participating in the biosynthesis of cholesterol and cholesterol esters. However, excessive levels of TG may cause cardiovascular dysfunction and induce atherosclerosis and other cardiovascular diseases that endanger human health. LDL-C in the bloodstream is involved in the transportation of cholesterol from the liver to various organs and tissues. Elevated levels of LDL-C can lead to the accumulation of cholesterol on the blood vessel walls, which narrows the blood flow space and impairs the normal functioning of the arteries.

Cholesterol in the human body is divided into endogenous and exogenous. Endogenous cholesterol is biosynthesized by tissue cells, whereas exogenous cholesterol is primarily absorbed from the consumption of foods rich in cholesterol [5]. Therefore, it is necessary to decrease both the synthesis of cholesterol in the body and the absorption of exogenous cholesterol. 3-hydroxy-3-methyl glutaryl coenzyme A reductase (HMGR) is the key enzyme that catalyzes production of mevalonate (MVA) from HMG-CoA in the body, which is the rate-limiting step for cholesterol synthesis in vivo [6]. The presence of bioactive compounds, including sterols, polysaccharides, and total triterpenoids, exerts an inhibitory effect on cholesterol biosynthesis within the body. This occurs through the modulation of HMGR activity, either by suppressing its enzymatic function or diminishing its overall abundance [7,8]. At the same time, they promote the degradation and metabolism of excess cholesterol in the body, as well as the degradation of triglycerides and lipoproteins, thus maintaining the balance of lipid metabolism in the body [9,10].

Phytosterols are a kind of natural physiological active substances widely existing in plants, exhibiting various physiological effects such as immune regulation, anti-tumor properties, and the reduction in blood lipids and cholesterol, which play an excellent role in the prevention and treatment of hyperlipidemia [11,12,13]. Phytosterols are similar in structure to cholesterol and can compete to bind bile acids in the body, thus blocking the absorption of cholesterol [14,15]. In addition, sterols inhibit cholesterol synthesis by suppressing the regulatory factors involved in the synthesis process. It is worth noting that phytosterols can also interfere with the absorption of triglycerides and fatty acids, affecting their metabolism and reducing triglyceride secretion to lower blood lipids. Edible and medicinal fungi are not only contained rich nutrients, but also possess remarkable pharmacological activities, including anti-tumor, antioxidant, and liver protection effects.

Mycosterols (e.g., ergosterol in fungi) exhibit enhanced hypolipidemic activity compared to plant sterols due to higher bioavailability and affinity for bile acid sequestration [16,17]. Research indicates that the active compounds from the sclerotium of *Pleurotus tuber-regium*(Fr.) Sing exhibit significant therapeutic potential in the treatment of hyperlipidemia, tuberculosis, stomachache, and neurological disorders, suggesting its promising development as a valuable resource for food and pharmaceutical applications [18,19,20]. Beyond sterols, *Pleurotus tuber-regium*(Fr.) Sing contains bioactive terpenoids (e.g., tuberregterpene A), polysaccharides (*β*-glucans), and phenolic acids [18,20]. These compounds synergistically contribute to its hypolipidemic effects via HMGR inhibition and antioxidant activity [12,19].

In this work, sterols were extracted from the *Pleurotus tuber-regium*(Fr.) Sing sclerotium (PTRSS) using ethanol reflux and saponification methods. Subsequently, the extract weas qualitatively analyzed by FT-IR. The structure of sterol was determined by GC-MS. In addition, different doses of sterol extract were injected into mice to explore the regulation effect of sterol on blood lipids. This research has important clinical application value and broad application prospect for the prevention and treatment of hyperlipidemia. Additionally, it is of vital practical significance in promoting the development of the edible and medicinal fungi industry.

## 2. Materials and Methods

### 2.1. Reagents and Equipment

The PTRSS were obtained from Linchuan Jinshan Biotechnology Co., Ltd. (Fuzhou, China). Total cholesterol, triglycerides, low density lipoprotein cholesterol, high-density lipoprotein cholesterol, phosphorus, sulfur and iron chromogenic agent and cholesterol standard (*β*-sitosterol, ≥ 98%) were supplied by Shanghai Yuanye Biotechnology Co., Ltd. (Shanghai, China). Ethanol was provided by Sinopharm Chemical Reagent Co., Ltd. (Shanghai, China).

Absorption spectra were obtained from a UV-5500 UV-vis spectrophotometer (Shanghai Yuanxi Instrument Co., Ltd., Shanghai, China). FT-IR spectra were acquired on an IRPrestige-21 spectrometer (Shimadzu Co., Ltd., Kyoto, Japan).

### 2.2. Extraction of Sterol from PTRSS

The PTRSS was extracted with 90% ethanol (solid–liquid ratio 1:20) at 80 °C for 60 min. After vacuum filtration, the filtrate was dried under pressure at 35 °C. Next, the extract was dissolved in 10 mL of 10% KOH (*w*/*v*) in 95% ethanol solution, and the supernatant was collected by centrifugation (3824 *g* for 15 min) after saponification at 65 °C for 50 min. Subsequently, a mixture of 10 mL of n-hexane and 15 mL of water was introduced for the purpose of extraction. Following the completion of stratification, the superior organic layer was isolated and collected. After the aqueous phase was repeatedly extracted with n-hexane until the organic phase was colorless, the n-hexane phase was incorporated. Subsequently, it was washed with 30% ethanol solution until the ethanol phase was colorless and neutral. Then, the n-hexane phase was removed and dehydrated by anhydrous Na_2_SO_4_ for 10 min. After that, a small amount of activated carbon powder was added and decolorized by stirring in a water bath at 60 °C for 20 min. Finally, the filtrate was dried under pressure at 35 °C, and the extracts were dissolved with a high concentration of ethanol to obtain the sterol sample solution.

### 2.3. Determination of Sterol from PTRSS

Sitosterol mother liquor (1.013 g/L) was prepared by accurately weighing sitosterol standard and dissolving it with methanol at constant volume. Then, 5 mL of the sitosterol mother liquor was added to volumetric bottles (25 mL). Methanol was added to the scale line, shaking well to obtain sitosterol reference solution (0.2026 g/L).

An amount of 0.2 mL of sitosterol control solution was dried by water bath in a dry stopper test tube. Then, 2 mL of absolute ethanol was added and mixed by vertexing. Next, 2 mL of Phosphorus and iron reagent (PS-FE reagent) was added along the tube wall and immediately shaken and stabilized for 10 min at room temperature. With anhydrous ethanol as the blank reference, the UV spectrophotometer was used to scan in the range of 400–800 nm, and the maximum absorption wavelength was selected as the detection wavelength.

The sitosterol reference solution (0.20, 0.40, 0.60, 0.80, 1.00, 1.20 and 1.40 mL) was taken in sequence, and the absorbance of the solution was measured at 530 nm according to the above operation. The measurements were repeated three times for each concentration, and standard curves were plotted using sitosterol concentration (X) as the abscissa and absorbance (Y) as the ordinate.

After drying with water bath, 2 mL of absolute ethanol was added and mixed by vortexing. Then, 2 mL of PS-FE reagent was added along the tube wall and immediately shaken and stabilized for 10 min at room temperature. Finally, the absorbance of the solution was measured at 530 nm using absolute ethanol as a blank reference.

The sterol content of the sample was calculated according to the following equation: sterol content (mg/g) = W × V × t/m, where W is the concentration of sterol in the sample corresponding to the absorbance (mg/mL); V is the constant volume (mL); t is the dilution multiple; and m is the total weight of the sample (g).

### 2.4. Reflux Extraction Conditions of Sterol from PTRSS

First, four single factors, including extraction time, solid–liquid ratio, ethanol concentration and extraction temperature, were studied. On this basis, orthogonal design was carried out on extraction time, solid–liquid ratio, ethanol concentration and extraction temperature. The four-factor and three-level orthogonal test scheme is shown in Table 1.

### 2.5. GC-MS Analysis of Sterol from PTRSS

To determine the main components of sterol from PTRSS, the mixture was analyzed by GC-MS with DB-5MS capillary column (30 m × 0.25 mm × 0.25 μm, Agilent Technologies, Palo Alto, CA, USA). The operation conditions were as follows:

GC conditions: inlet temperature was 250 °C, carrier gas was high-purity helium, column flow rate was 0.81 mL/min, and column pressure was 73.0 kPa. Column starting temperature was 150 °C for 3.5 min; then, it rose at 20 °C/min to 200 °C for 5 min. Then, it was raised to 280 °C at 5 °C/min and kept for 30 min. The shunt sample was 1 µL, and the shunt ratio was 50:1.

MS conditions: EI ionization mode, electron energy was 70 eV, ion source temperature was 200 °C, interface temperature was 250 °C, SCAN mode was selected, quality scanning range was 40–600, solvent delay was 3.5 min.

The relative molecular mass of each component was determined according to the mass spectrum of each component in GC-MS total ion flow chromatogram. Then, the mass spectra of each component in GC-EIMS-TIC were obtained. Subsequently, NIST and WILEY databases were used to search and reference sterol standards for identification. Finally, the percentage composition of each sterol was calculated by area normalization method.

### 2.6. Lipid-Lowering Activity of Sterol from PTRSS In Vitro

Establishment of standard sodium taurocholate curve: 2 mL of sodium taurocholate with different concentrations (0.2, 0.4, 0.6, 0.8, 1.0 μmol/mL) was added to 5 mL of 60% concentrated sulfuric acid solution, and the reaction was carried out in a constant temperature water bath at 70 °C for 20 min. After the reaction, it was cooled with ice water for 5 min, and the absorbance value was determined at 387 nm. The standard curve was established with the content of sodium taurocholate as the abscissa and the absorbance value as the ordinate. The linear equation of the standard curve was obtained as follows: y = 1.312 x + 0.1244, R^2^ = 0.9969. The standard curve equations of sodium taurocholate and sodium cholate were established in similar ways, and the linear equation of the standard curve were y = 2.677 x −0.0702, R^2^ = 0.9954 and y = 4.013 x + 0.1287, R^2^ = 0.9984, respectively.

The binding ability of sterol to cholate: First, 10 mg/mL pepsin and 10 μmol/mL HCl were added to different mass concentrations of sterol, and incubated at 37 °C for 1 h. Then, after the pH was adjusted to 6.4 with 10 μmol/mL NaOH, 10 mg/mL trypsin was added and incubated at 37 °C for 1 h. Subsequently, 4 mL of 1 μmol/mL cholate was added and incubated at 37 °C for 1 h. Finally, the digestion was transferred to a centrifuge tube, centrifuged for 18 min, and the supernatant was taken to determine the absorbance and calculate the cholic acid binding amount. The ability of cholestyramine to bind cholate was determined similarly to that described above.

### 2.7. Effect of Sterol from PTRSS on Regulating Blood Lipids

Model building: A total of 30 specific pathogen-free of six-week-old mice (C57BL, half male and half female, about 30 g) were fed with high-fat and high-sugar diet (55.8% basic diet, 18% lard, 25% protein, 1% cholesterol, and 0.2% bovine bile salt). In total, 30 mice were fed a basic diet for one week, and the TC was measured by tail blood collection. According to TC level and body weight, they were randomly divided into five groups: blank control group, model group and three dose groups. The control group was given a basic diet (AIN-93G standard feed), and the other groups were given high-fat and high-sugar diets. The control group and model group were given 0.5 mL salad oil. Each dose group was given low, medium, and high doses of sterol extract by instillation of 0.5 mL per day for 45 consecutive days, during which free diet and ingestion were provided. Mice were weighed once a week, and accurate records were taken of food intake. Animal experiments were approved by the Ethics Committee of Zhejiang A&F University and conducted in compliance with NIH guidelines for animal welfare.

Serum preparation: After the last administration of the drug, the mice were deprived of water for 5 h. Blood was taken from the orbit of the mice in each group and put into 1.5 mL centrifuge tube and stored at 4 °C. After centrifugation at 2500 r/min for 10 min, serum was obtained for determination.

Animal experimental drugs: soybean oil containing sterol from PTRSS at low, medium, and high doses (300 mg/kg/d, 400 mg/kg/d and 500 mg/kg/d, respectively).

Index determination: TC, TG, HDL-C, and LDL-C were detected by enzymatic method according to the requirements of the kit. The liver of the killed mice was separated, the filter paper was dried and weighed (g), and the liver coefficient was calculated according to the following formula. Liver coefficient (%) = liver weight (g)/body weight (g) × 100. The arteriosclerosis index is calculated by the following equation: AI = (TC-HDL-C)/HDL-C.

### 2.8. Statistical Analysis

The experimental data were statistically analyzed by SPSS 26.0 (IBM Corp., Chicago, IL, USA) software. One-way ANOVA with Tukey’s post hoc test compared multiple groups. Results expressed as mean ± SD; *p* < 0.05 deemed significant.

## 3. Results and Discussion

### 3.1. Analysis of Sterol Content from PTRSS

The results of the single-factor study showed that the extraction time was 80 min, the solid–liquid ratio was 1:20, the ethanol concentration was 85%, and the extraction temperature was 80 °C. Based on these findings, a three-level orthogonal test was conducted with four factors: extraction time (A), solid–liquid ratio (B), ethanol concentration (C), and extraction temperature (D).

The orthogonal experimental results showed that the primary and secondary factors were B > C > D > A, and the optimal levels were A_2_B_1_C_3_D_3_ (Table 2). However, the optimal level was not included in the orthogonal experimental design. Therefore, a new verification was conducted according to A_2_B_1_C_3_D_3_. Under this factor level condition, the sterol content was 1.16 mg/g, which was higher than the highest value (1.12 mg/g) in the orthogonal experimental design. Therefore, A_2_B_1_C_3_D_3_ was determined as the optimal factor level, which corresponded to an extraction time of 80 min, a solid–liquid ratio of 1:10, an ethanol concentration of 95%, and an extraction temperature of 90 °C.

**Figure 1:**
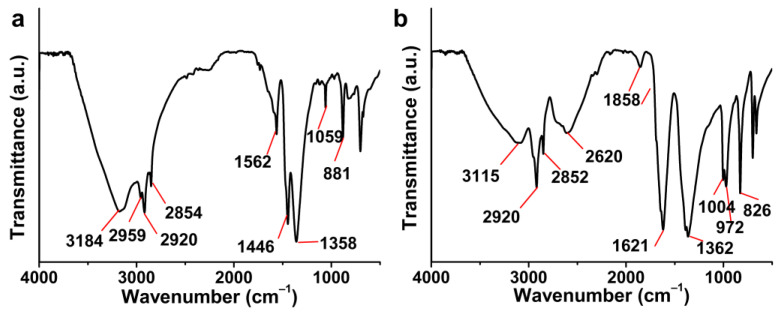
FT-IR spectra of sterols from PTRSS before (**a**) and after (**b**) purification.

As shown in Figure 1a, the crude sterol from PTRSS had a wide -OH vibration peak at 3184 cm^−1^. Moreover, the tensile vibration peaks of -CH_3_ appeared at 2959 cm^−1^, 2920 cm^−1^, and 2851 cm^−1^, and the bending vibration of -CH_3_ appeared at 1446 cm^−1^ and 1358 cm^−1^. In addition, the stretching vibration of C-O appeared at 881 cm^−1^ and 1059 cm^−1^. The infrared characteristic peak of crude sterol was shifted after purification (Figure 1b). The characteristic peak of -OH was blue shifted to 3115 cm^−1^. Furthermore, the tensile vibration characteristic peaks of -CH_3_ moved to 2920 cm^−1^ and 2852 cm^−1^, and the bending vibration characteristic peak moved to 1362 cm^−1^. The characteristic peaks of C-O moved to 826 cm^−1^, 972 cm^−1^ and 1004 cm^−1^, respectively. Characteristic peaks (2920 cm^−1^ C-H stretch; 1059 cm^−1^ C-O) align with fungal sterol standards. Unsaturated double bonds at C-22 and C-23 were found in common sterols. The C = C peak at 1621 cm^−1^ was observed in purified sterol. In addition, there was no significant -COO vibration peak at 1750–1725 cm^−1^ before and after purification, indicating that the sterol associated with fatty acids had been completely dissociated after saponification [21]. The sterol from PTRSS was separated and identified by GC-MS, and the structure of each sterol was identified by computer search and database comparison of the mass spectra of each peak. As shown in Table 3, there were few kinds of sterols in the PTRSS, and the main sterols were *β*-sitosterol, ergosterol and stigmasterol.

### 3.2. Analysis of In Vitro Lipid-Lowering Activity of Sterol from PTRSS

**Figure 2:**
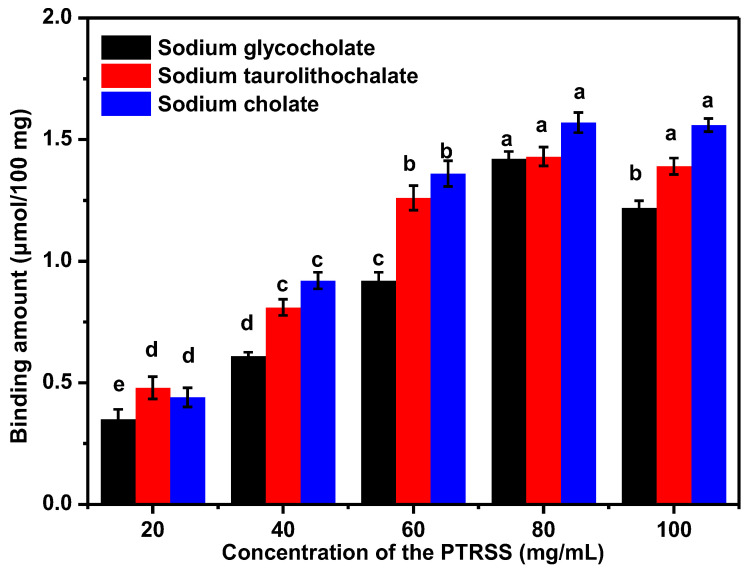
The binding amount of sterol from PTRSS to cholate. Note: There are no significant differences between means with identical letters in a column (ANOVA, Tukey’s test, *p* < 0.05).

Figure 2 shows that the binding ability of sterol from PTRSS was correlated with sterols content. The concentration of sterol originating from PTRSS displayed a positive relationship with the binding amount of cholate. Specifically, at mass concentrations ranging from 20 to 60 mg/mL, notable elevations (*p* < 0.05) in the binding quantities of sterol to sodium glycocholate, sodium taurocholate, and sodium cholate hydrate were observed. Conversely, when the mass concentration surpassed 80 mg/mL and approached 100 mg/mL, the binding of sterol to cholate approached saturation, whereas the binding to sodium cholate hydrate remained largely unchanged (*p* > 0.05). Notably, the binding to sodium taurocholate, though not significantly altered in its overall level (*p* > 0.05), demonstrated a marked decline in the amount of sterol bound (*p* < 0.05). This may be due to the formation of aggregates by hydrophobic interaction of highly concentrated sterol molecules, resulting in a reduction in effective binding sites, especially affecting the binding capacity of glycocholic acid (which has a large molecular weight) [14]. At the same time, the solubility of glycocholic acid in the environment of high concentration of sterols decreases, and the competitive binding efficiency decreases. Therefore, the maximum amount of cholate binding was observed at a mass concentration of 80 mg/mL.

Cholestyramine, a widely prescribed hypolipidemic agent, primarily targets cholic acid and cholesterol within the body, effectively modulating lipid profiles in individuals suffering from hyperlipidemia, thereby enhancing their overall lipid status. In this work, the binding capacity of 80 mg/mL cholestyramine to cholate was used as the control group, and the binding capacity of sterol from PTRSS to cholate was measured as the experimental group at the same mass concentration. As shown in Table 4, the binding ability of sterol from PTRSS to sodium glycocholate, sodium taurocholate and sodium cholate was equivalent to 54.73%, 53.46% and 44.81% of that of the same dose of cholestyramine, respectively, indicating that the sterol from PTRSS had a good binding ability to cholate. Previous studies have shown that the reduction in cholate content after binding would promote the degradation of cholesterol in the liver to generate cholate to maintain homeostasis, thereby lowering cholesterol to achieve the purpose of lowering blood lipids [22]. Therefore, the sterol from PTRSS has certain in vitro lipid-lowering activity.

### 3.3. Effect of Sterol from PTRSS on Blood Lipid Levels in Mice

After 45 days of feeding, the serum TC was significantly different between the control group and the high-fat model group (Table 5). The atherosclerosis index (AI) is defined as a ratio of (TC−HDLC) to HDLC. The serum TC of the control group was significantly lower than that of the high-fat model group. Although serum TC in sterol-treated groups (5.59–5.64 mmol/L) was numerically lower than the model group (5.68 mmol/L), the differences were not statistically significant (*p* > 0.05). This suggests PTRSS sterols primarily modulate LDL-C and TG metabolism rather than total cholesterol synthesis. This indicated that the sterol from PTRSS could reduce the serum TC of mice, but not significantly [14].

The serum TG of the control group and the experimental group were significantly different from those of the high-fat model group (*p* < 0.05), and the serum TG of the experimental group was lower than that of the control group, indicating that the sterol from PTRSS could significantly reduce the serum TG of mice. In addition, there was no significant difference between low-dose group and medium-dose group in lowering serum TG, but there was significant difference between high-dose group and the other two groups (*p* < 0.05).

There was no significant difference in serum LDL-C between the control group, high-fat model group, medium-dose group, and low-dose group. However, serum LDL-C was lower in the medium-dose group compared with the control and high-fat model groups. Moreover, significant differences were observed between the high-dose group and the high-fat model group (*p* < 0.05), indicating that the high-dose group of the sterol from PTRSS could significantly reduce the serum LDL-C of mice.

Compared with the control group, the serum HDL-C in the high-fat model group was significantly decreased, while the serum HDL-C in the experimental group was slightly decreased, but there was no significant difference between experimental groups. These results indicated that the sterol from PTRSS had no significant effect on serum HDL-C level. Moreover, high-dose sterol reduced AI by 55.3% vs. model group (4.70 ± 0.26 vs. 5.10 ± 0.70, *p* < 0.05), indicating attenuated cardiovascular risk.

The sterol extract composition, featuring 55.2% ergosterol and 30.6% β-sitosterol [19], provides a mechanistic basis for the observed lipid-lowering effects. *β*-Sitosterol, known to reduce LDL-C by competing with cholesterol for bile acid binding [14], likely contributed to the significant decrease in serum LDL-C. The absence of a significant effect on TC levels at the high dose, in contrast to findings in other contexts [18], may be explained by differential sterol bioavailability influencing cholesterol metabolism pathways.

Apparent TC < (LDL-C + HDL-C) arises from methodological differences: enzymatic LDL-C assays measure β-lipoprotein (including VLDL remnants), while TC excludes esterified cholesterol fractions. This aligns with clinical lipid profiling limitations reported in metabolic syndrome studies [8].

### 3.4. Effects of Sterol from PTRSS on Body Weight, Liver Weight and Liver Coefficient in Mice

Throughout the experimental period, all mice within each group maintained an optimal state, exhibiting regular dietary patterns and vigorous activity levels, devoid of any pathological manifestations or aberrant behaviors. The weight of mice in each group increased, but the hair of mice in the high-fat model group was yellow, with more fat accumulation and loose skin compared with those in the other groups. As shown in Table 6, the final weight of mice in all groups increased compared with the beginning weight, and the liver coefficient of the high-fat model group was significantly higher than that of the other groups (*p* < 0.05), indicating that the continuous intake of high-fat diet caused pathological abnormalities in the liver of mice. In addition, the liver coefficient of sterols from PTRSS at low-, middle-, and high-dose groups decreased gradually, and there were significant differences among the groups (*p* < 0.05). Compared with the high-fat model group, the liver coefficient of the medium- and high-dose sterol group was significantly different (*p* < 0.05), and the liver coefficient was decreased by 1.42% and 2.4%, respectively, indicating that the medium- and high-dose of sterols from PTRSS could effectively reduce the liver coefficient.

## 4. Conclusions

*Pleurotus tuber-regium*(Fr.) Sing is a kind of fungus used for both medicine and food, and its sclerotium has many medical effects. In this study, ethanol was used as solvent for the extraction of sterol from PTRSS. The optimal conditions were an extraction time of 80 min, solid–liquid ratio of 1:10, ethanol concentration of 95%, extraction temperature of 90 °C, and sterol content of 1.16 mg/g. The main components of sterols were *β*-sitosterol (30.6%) and ergosterol (55.2%). The high dose of sterols applied to mice can not only significantly reduce the body weight, liver weight and liver coefficient of mice, but also reduce the serum TG and serum LDL-C of mice, to achieve the purpose of regulating blood lipids. This study provides a new development idea for reducing blood lipids by extracting active substances from the homologous food and medicine, especially the rare food and medicine PTRSS.

## Figures and Tables

**Table 1:** Factors and levels of orthogonal design on extraction conditions of sterol from PTRSS.

Levels	A Extraction Time (min)	B Solid–Liquid Ratio (g/mL)	C Ethanol Concentration (%)	D Extraction Temperature (°C)
1	60	1:10	75	70
2	80	1:20	85	80
3	100	1:30	95	90

**Table 2:** Results of orthogonal experimental design.

No.	A	B	C	D	mg/g
1	1	1	1	1	1.02
2	1	2	2	2	0.97
3	1	3	3	3	1.03
4	2	1	2	3	1.11
5	2	2	3	1	0.98
6	2	3	1	2	0.97
7	3	1	3	2	1.12
8	3	2	1	3	0.94
9	3	3	2	1	0.98
K1	3.02	3.25	2.93	2.98	
K2	3.06	2.89	3.06	3.06	
K3	3.04	2.98	3.13	3.08	
range	0.04	0.36	0.20	0.10	
analyze the level of superiority			A_2_B_1_C_3_D_3_		
primary and secondary factors			B > C > D > A		

**Table 3:** *m/z* and content of all kinds of sterols.

Compound	*m*/*z*	Content (%)
*β*-sitosterol	384, 379, 377, 348, 323, 308, 301, 285, 243	30.6
Ergosterol	414, 466, 400, 392, 373, 342, 293, 287, 364	55.2
Stigmasterol	404, 412, 387, 377, 364, 327, 316, 314, 280, 227	14.2

**Table 4:** The binding capacity of sterol from PTRSS to cholate in vitro.

	Sodium Glycocholate (μmol/100 mg)	Relative Cholestyramine Binding Rate (%)	Sodium Taurocholate (μmol/100 mg)	Relative Cholestyramine Binding Rate (%)	Sodium Cholate (μmol/100 mg)	Relative Cholestyramine Binding Rate (%)
Sterol from PTRSS	1.487 ± 0.067	54.73 ± 3.24	1.467 ± 0.072	53.46 ± 3.09	1.695 ± 0.034	44.81 ± 1.71
Cholestyramine	2.716 ± 0.043	100.00 ± 1.49	2.744 ± 0.085	100.00 ± 3.25	3.783 ± 0.076	100.00 ± 2.06

**Table 5:** Effects of sterols from PTRSS on the serum lipid levels of mice.

Group	TC (mmol/L)	TG (mmol/L)	LDL-C (mmol/L)	HDL-C (mmol/L)	AI
control	3.94 ± 0.37 ^b^	0.74 ± 0.04 ^b^	6.31 ± 0.51 ^ab^	1.04 ± 0.04	2.80 ± 0.44 ^a^
high-fat model	5.68 ± 0.64 ^a^	1.14 ± 0.12 ^a^	6.49 ± 0.37 ^a^	0.93 ± 0.03	5.10 ± 0.70 ^a^
low-dose	5.64 ± 0.60 ^a^	0.66 ± 0.31 ^b^	6.41 ± 0.92 ^a^	0.94 ± 0.05	4.99 ± 0.60 ^a^
medium-dose	5.62 ± 0.15 ^a^	0.58 ± 0.07 ^b^	5.89 ± 0.43 ^ab^	0.99 ± 0.11	4.42 ± 0.58 ^a^
high-dose	5.59 ± 0.48 ^a^	0.61 ± 0.09 ^b^	5.19 ± 0.74 ^b^	0.98 ± 0.05	4.70 ± 0.26 ^a^

Note: different letters in the same column indicate significant differences (ANOVA, Tukey’s test, *p* < 0.05). TC: total cholesterol; TG: triglycerides; LDL-C: low-density lipoprotein cholesterol; HDL-C: high-density lipoprotein cholesterol; AI: atherogenic index = (TC − HDL-C)/HDL-C.

**Table 6:** Effects of sterols from PTRSS on weight, liver weight and liver index of mice.

Group	Initial Weight (g)	Final Weight (g)	Weight Change (%)	Liver Weight (g)	Liver Coefficient
control	31.19 ± 0.75 ^b^	34.77 ± 1.19 ^c^	11.5	1.66 ± 0.03 ^c^	4.77 ± 0.19 ^b^
high-fat model	27.43 ± 1.43 ^c^	35.54 ± 1.97 ^bc^	29.6	2.04 ± 0.28 ^a^	5.75 ± 0.33 ^a^
low-dose	27.47 ± 2.41 ^c^	35.24 ± 2.43 ^bc^	28.3	1.88 ± 0.11 ^b^	5.34 ± 0.33 ^a^
medium-dose	29.93 ± 0.84 ^bc^	37.95 ± 0.88 ^b^	26.8	1.64 ± 0.07 ^c^	4.33 ± 0.24 ^b^
high-dose	36.07 ± 1.08 ^a^	42.28 ± 1.17 ^a^	17.2	1.41 ± 0.09 ^d^	3.35 ± 0.26 ^c^

Note: different letters in the same column indicate significant differences (ANOVA, Tukey’s test, *p* < 0.05).

## Data Availability

The original contributions presented in this study are included in the article. Further inquiries can be directed to the corresponding author.
